# Benefits and risks associated with different uses of the COVID-19 vaccine Vaxzevria: a modelling study, France, May to September 2021

**DOI:** 10.2807/1560-7917.ES.2021.26.26.2100533

**Published:** 2021-07-01

**Authors:** Cécile Tran Kiem, Alessio Andronico, Paolo Bosetti, Juliette Paireau, Lise Alter, Pierre-Yves Boëlle, Arnaud Fontanet, Daniel Lévy-Bruhl, Simon Cauchemez

**Affiliations:** 1 Mathematical Modelling of Infectious Diseases Unit, Institut Pasteur, UMR2000, CNRS, Paris, France; 2 Collège Doctoral, Sorbonne Université, Paris, France; 3 Santé Publique France, French National Public Health Agency, Saint-Maurice, France; 4 Haute Autorité de Santé, Saint-Denis la Plaine, France; 5 Institut Pierre Louis d’Epidémiologie et de Santé Publique, Sorbonne Université, INSERM, Paris, France; 6 Emerging Diseases Epidemiology Unit, Institut Pasteur, Paris, France; 7 PACRI Unit, Conservatoire National des Arts et Métiers, Paris, France

**Keywords:** SARS-CoV-2, vaccine, thrombosis, AstraZeneca, Vaxzevria, benefit-risk balance

## Abstract

Thrombosis with thrombocytopenia (TTS) has been identified as a rare adverse event following COVID-19 vaccination with Vaxzevria. We modelled the benefits and risks of Vaxzevria distribution from May to September 2021 in metropolitan France where other vaccines are available, considering French hospitalisation data and European data on TTS. Across different scenarios, benefits of Vaxzevria distribution in people 55 years and older exceeded the risk of death from COVID-19. In young adults, risks were at least of similar magnitude as benefits.

On 7 April 2021, the European Medical Agency (EMA) concluded that a causal relationship between vaccination with Vaxzevria (*ChAdOx1-S;* AstraZeneca, Cambridge, United Kingdom [1]; previously named Oxford–AstraZeneca COVID-19 vaccine) and adverse events of thrombosis in combination with thrombocytopenia (TTS) was at least a reasonable possibility [2]. Evaluating the balance of benefits and risks associated with different distribution strategies for Vaxzevria is of paramount importance to maximise health benefits and maintain trust in vaccination. We used a mathematical model to evaluate this, accounting for both the indirect effect of vaccination and the availability of alternative vaccines, using metropolitan France to illustrate the situation of European countries that are at a comparable stage in their vaccination campaign.

## Modelling the impact of Vaxzevria distribution strategies

We used an age-stratified compartmental model describing the spread of severe acute respiratory syndrome coronavirus 2 (SARS-CoV-2) in the population of metropolitan France [3]. Modelling assumptions are described in detail elsewhere [4] and are summarised in the Supplement. The model accounted for the emergence of the more transmissible and severe Alpha variant (hereafter referred to using the Phylogenetic Assignment of Named Global Outbreak (Pango) lineage designation B.1.1.7) as well as the progressive roll-out of vaccines [4]. In the following, we denote by historical lineages strains that were circulating in France in 2020. We did not account for the circulation of variants others than B.1.1.7. The model was calibrated on daily hospital admissions reported in the national SI-VIC surveillance system (the information system for the monitoring of victims of terror attacks and exceptional sanitaries situations - Système d'information pour le suivi des victimes d'attentats et de situations sanitaires exceptionnelles (Covid-19)) [5] and communicated by Santé Publique France, the French national public health agency, and on the proportion of B.1.1.7 among positive RT-PCR tests over time.

We assumed that mRNA vaccines (Comirnaty, BioNTech/Pfizer, Mainz, Germany/New York, United States (US) [6] and Spikevax, Moderna, Cambridge, US [7]) are used in the entire adult population (18 years and older) and that the viral vector COVID-19 vaccine Janssen (Ad26.COV2-S (recombinant), Janssen-Cilag International NV, Beerse, Belgium) [8] is only used in people 55 years and older, in line with current French recommendations. We explored different distribution strategies for Vaxzevria from 8 May: (i) to the entire adult population, (ii) to those at least 40 years-old or (iii) to those at least 55 years-old. Vaccination starts in a younger age group when the target vaccine coverages are reached in groups of higher priority (see Supplement). We considered target vaccine coverages of 85% in individuals 65 years and older and 70% in individuals aged 18–64 years.

**Figure 1:**
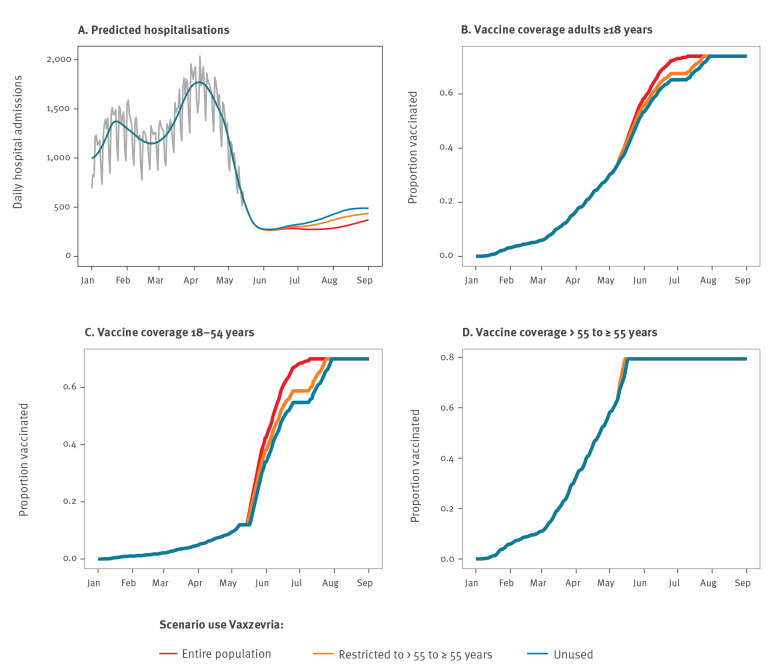
Predicted impact of different uses of the COVID-19 Vaxzevria vaccine on the daily hospital admissions and overall COVID-19 vaccine coverage, France, January–September 2021

In our baseline scenario, we assumed that all vaccines are 90% effective against severe forms of coronavirus disease (COVID-19) and 80% effective against infection [1,6,7,9,10], that B.1.1.7 is 60% more transmissible than historical lineages circulating in 2020 and that the progressive relaxation of measures implemented on 19 May 2021 will increase the intervention reproduction number R_I_ of the historical viruses to 1.2 from that date on, and to 1.3 after 1 July. The R_I_ is the average number of persons infected by a case under a given set of control measures if there is no immunity in the population. By 7 May 2021, 25.7% of the population in metropolitan France had received a first vaccine dose [11], and we assumed that from 8 May 2021, mRNA vaccines can be rolled out at a pace of 500,000 doses per day altogether and the viral vector Janssen and Vaxzevria vaccines at 100,000 doses per day each. Finally, we assumed that 19.3% of the population had been infected by SARS-CoV-2 by 7 May 2021. Vaccine coverage by age is shown in Figure 1.

COVID-19: coronavirus disease.

Panel A shows the predicted daily hospital admissions over time in our baseline scenario assuming an intervention reproduction number R_I(19 May–1 July)_ of 1.2 for the historical virus, panels B–D show the predicted vaccination coverage for different uses of the Vaxzevria vaccine. The grey line in panel A corresponds to hospital data reported in the SI-VIC surveillance system [5] used for model calibration.

For each Vaxzevria distribution strategy, we computed (i) the number of admissions to an intensive care unit (ICU) and deaths averted in the different age groups compared with a scenario where Vaxzevria is no longer distributed and (ii) the expected number of ICU admissions and deaths from TTS based on risks estimated by the EMA (Supplementary Table S1) [12]. The latter assessment was performed assuming that 100% of TTS cases will be admitted to the ICU and 30% will die [13,14].

## Population impact of Vaxzevria distribution strategies

In the scenario where there is no restriction on the use of Vaxzevria among adults, we expected 38,100 COVID-19 hospitalisations between 8 May and 1 September 2021 (Figure 1A). This number would increase to 42,400 if the use was restricted to people 55 years and older and to 45,900 if the use was stopped. If the use of Vaxzevria was discontinued, the time to reach the target vaccine coverage (85%) in those 55 years and older would be delayed by only a few days, whereas it could take up to 20 days more to reach the vaccine coverage of 70% for those aged 18–54 years (Figure 1B-D).

## Balance of risks and benefits associated to the use of Vaxzevria

**Figure 2:**
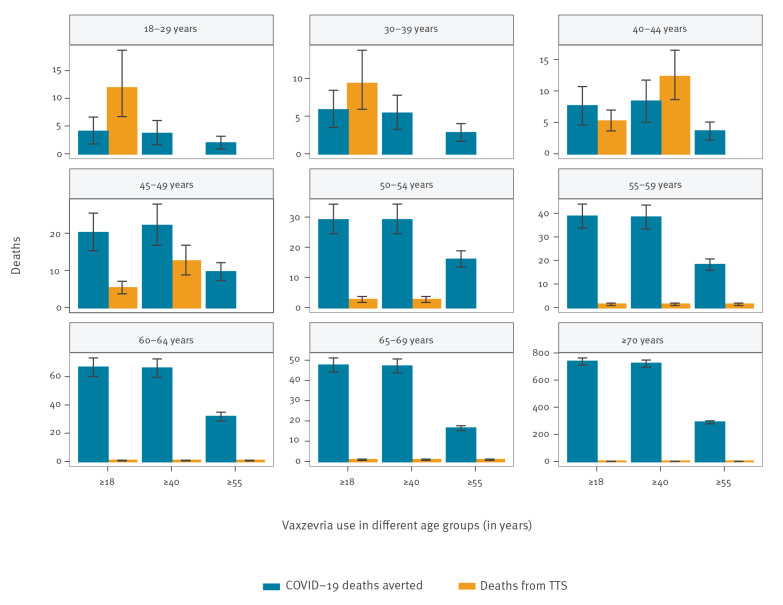
Predicted number of averted COVID-19 deaths and Vaxzevria-related TTS deaths for different uses of Vaxzevria compared with a strategy where the vaccine is not used, France, 8 May–1 September 2021

COVID-19: coronavirus disease; TTS: thrombosis in combination with thrombocytopenia.

In all distribution strategies, the number of COVID-19 deaths averted with the use of Vaxzevria in individuals 55 years and older was substantially higher than the expected number of deaths from TTS in that age group (Figure 2). For instance, using Vaxzevria in those 55 years and older would avert 355 (95% prediction interval (PI): 337–373) deaths in this group while causing three (95% PI: 2–5) deaths from TTS, compared with the scenario of discontinuation. When Vaxzevria was used in younger age groups, the benefit–risk balance was no longer as favourable and even reversed in the younger age groups. For instance, using Vaxzevria in the entire adult population would avert four (95% PI: 2–7) COVID-19 deaths in the 18–29 year-olds and six (95% PI: 3–8) in the 30–39 year-olds, but it would be associated with 12 (95% PI: 7–19) and nine (95% PI: 6–14) deaths from TTS in these age groups, respectively.

Three distribution strategies for Vaxzevria are explored: (i) in the entire adult population (≥ 18), (ii) in people 40 years and older (≥ 40) and (iii) in those 55 years and older (≥ 55). The vertical error bars correspond to 95% prediction intervals accounting for the uncertainty in the risk of TTS following vaccination with Vaxzevria and in the risk of death following hospitalisation in the different age groups.

**Figure 3:**
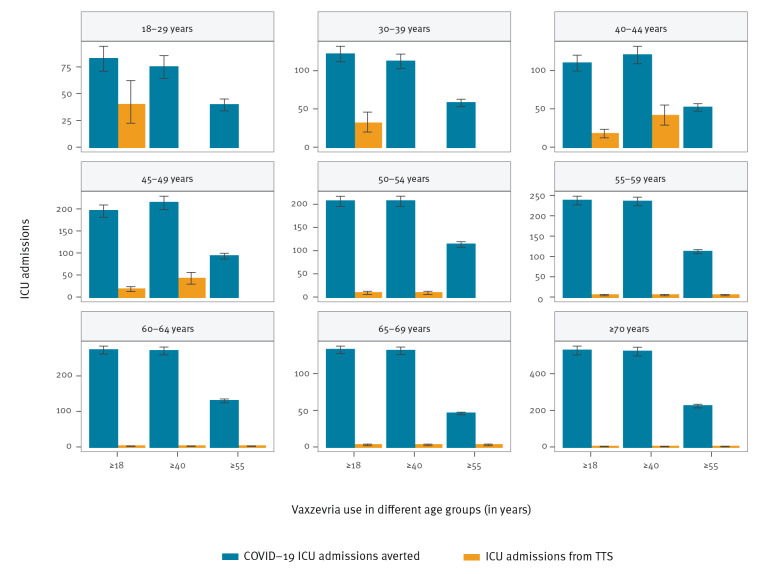
Predicted number of averted COVID-19 ICU admissions and Vaxzevria-related TTS ICU admissions for different uses of Vaxzevria compared with a strategy where the vaccine is not distributed, France, 8 May–1 September 2021

COVID-19: coronavirus disease; ICU: intensive care unit; TTS: thrombosis in combination with thrombocytopenia.

The number of COVID-19 ICU admissions averted with the use of Vaxzevria remained larger than ICU admissions for Vaxzevria-related TTS, in all age groups and all strategies of use for Vaxzevria (Figure 3). This is explained because in younger age groups, the risk of ICU admission following infection is higher than the risk of death following infection. 

Three distribution strategies for Vaxzevria are explored: (i) in the entire adult population (≥ 18), (ii) restricted to those 40 years and older (≥ 40 years), (iii) restricted to those 55 years and older (≥ 55). The vertical error bars correspond to 95% prediction intervals accounting for the uncertainty in the risk of TTS following vaccination with Vaxzevria and in the risk of death following hospitalisation in the different age groups.

## Sensitivity analyses

**Figure 4:**
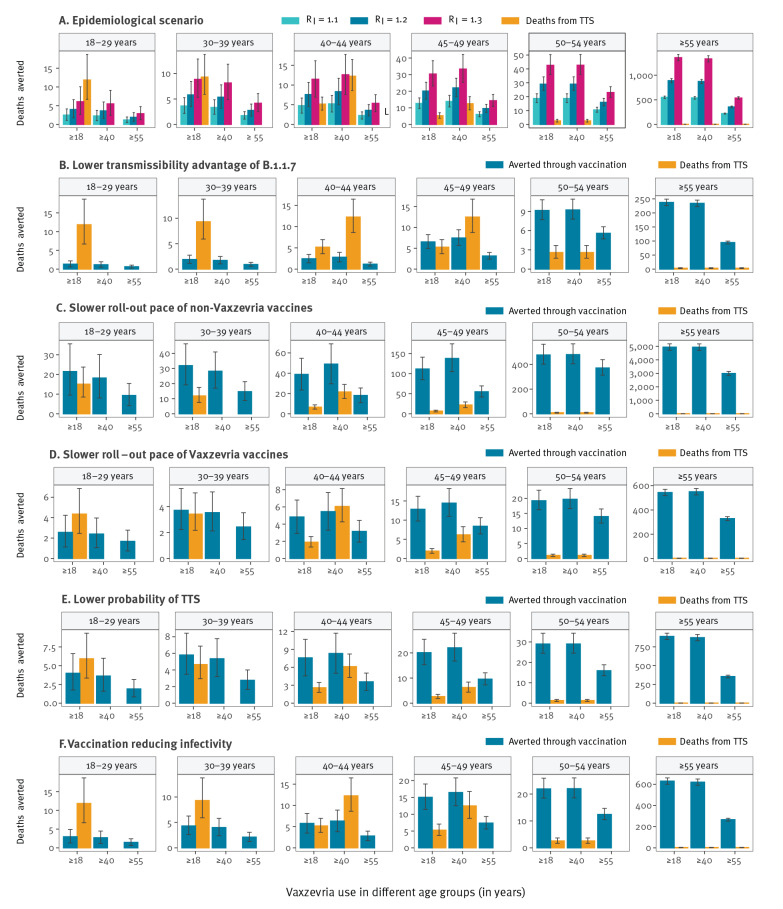
Sensitivity analyses on the benefits and risks in terms of deaths for different uses of Vaxzevria, France, 8 May–1 September 2021

COVID-19: coronavirus disease; TTS: thrombosis in combination with thrombocytopenia.

We performed a range of sensitivity analyses to understand how these assessments may change when varying the epidemiological scenario, the transmissibility advantage of B.1.1.7, the roll-out pace of Vaxzevria and non-Vaxzevria vaccines, the risk of TTS in vaccinated individuals and the effect of vaccines on transmission (Figure 4). In all sensitivity analyses, the number of deaths averted in individuals 55 years and older with vaccination in the different Vaxzevria distribution strategies was always substantially higher than the expected number of deaths from TTS (Figure 4). In young adults, the balance of benefits and risks for death is never favourable. In individuals aged 40–54 years, the ranking between risks and benefits depended on assumptions regarding roll-out and epidemic dynamics.

Panel A explores different values of the intervention reproduction number R_I_ between 19 May and 1 July 2021. Panel B assumes an increased transmissibility of 40% for B.1.1.7 compared with the historical virus. Panel C assumes a slower roll-out pace of mRNA vaccines (100,000 doses per day for mRNA vaccines). Panel D assumes a slower roll-out pace of Vaxzevria (50,000 doses per day). Panel E assumes a lower probability of TTS (risk of TTS per vaccine and not per dose). Panel F assumes that vaccinated individuals are half as infectious as unvaccinated individuals. The vertical error bars correspond to 95% prediction intervals accounting for the uncertainty in the risk of TTS following vaccination with Vaxzevria and in the risk of death following hospitalisation in the different age groups.

## Ethical statement

Ethical approval was not required for this analysis, which was based on aggregated hospitalisation and test data as well as TTS risk estimates communicated by the EMA. 

## Discussion

We found that, for individuals 55 years and older, the benefits of distributing Vaxzevria largely outweighed the risks in a range of possible scenarios. In contrast, in young adults, the risks were similar or higher than the benefits. These conclusions were driven by the steep increase in the severity of infection with age [15] as well as the higher risk of TTS following vaccination in individuals younger than 50 years [12]. The vaccination of older individuals with Vaxzevria reduced the number of deaths in this age group due to both direct (i.e. vaccinated individuals have a lower probability of fatal outcome) and indirect protection (i.e. younger adults who play a substantial role in transmission will be vaccinated earlier, reducing the risk of infection in all age groups). 

We relied on dose availability and distribution capacities in France, but because of the joint procurement mechanism, our conclusions should be of relevance for other European Union countries. In other settings with lower availability of vaccines doses other than Vaxzevria, the impact of restricting its distribution on the number of deaths averted could be much larger. Our assessments were strongly influenced by assumptions regarding vaccine roll-out but were based on delivery volumes anticipated in May 2021. We assumed that the number of unused Vaxzevria doses would not be replaced by other vaccines, i.e. that stopping the roll-out of Vaxzevria would not result in an increase in the roll-out pace of other vaccines (non-fungible distribution channels). Should this change in the coming months (e.g. with the storage of mRNA vaccines at higher temperatures facilitating this distribution), benefits associated with the distribution of Vaxzevria would decrease substantially. French residents are also increasingly reluctant to get vaccinated with this vaccine, and the number of doses of Vaxzevria used has plateaued at around 30,000–50,000 per day throughout May and June 2021 while it has increased for mRNA vaccines (Supplementary Figure S1) [11]. If the vaccine is not used much, both benefits and risks associated with it will be limited.

Our assessment relies on estimates of the risk of TTS calculated by the EMA [12] which might underestimate risks as they are based on reported cases. The latter estimates are however higher than those estimated in the United Kingdom based on the yellow cards reports from the Medicines and Healthcare products Regulatory Agency (Supplementary Figure S2) [16]. Other elements which we do not account for, including the spread of variants such as B.1.351 for which Vaxzevria may be less effective [17], would reduce the benefits associated with its distribution. The rise to dominance of the Delta variant is expected to complicate epidemic control. However, in the context of France and a number of European countries, this may happened during summer (at the end of our study period) so that this should only have a limited impact on our results. 

Comparing numbers of deaths or ICU admissions induced and averted by Vaxzevria cannot capture all dimensions of the decision regarding vaccination both at individual and population level. Such a decision should weigh the different natures of involved risks: on the one side, a potential severe side effect following a preventive intervention and on the other side, an hypothetical risk of disease complications within an unknown time horizon. Providing accurate risk–benefit scenarios is crucial, but is not enough to ensure compliance with vaccination [18].

## Conclusion

This analysis provides, across a range of scenarios, a quantitative assessment of the balance between risks and benefits associated with different uses of the COVID-19 vaccine Vaxzevria, accounting for the indirect effect of vaccination as well as the availability of alternative vaccines. Our results highlight the clear benefit of the distribution of Vaxzevria towards the population aged 55 years and older and provide valuable insight for public health decision making. 

## Supplementary Data

569000 Supplement
