# Supplementary material for: Benefits and risks associated with different uses of the COVID-19 vaccine Vaxzevria: a modelling study, France, May to September 2021
Source: Euro Surveill. 2021 Jul 1;26(26):2100533. doi: 10.2807/1560-7917.ES.2021.26.26.2100533 (PMC8326661; doi:10.2807/1560-7917.ES.2021.26.26.2100533)
Supplement: Supplement [file 21-00533_Supplement_Cauchemez.pdf]

Supplement for: Benefits and risks associated with different uses of the Vaxzevria vaccine: a modelling study

Cécile Tran Kiem MSc[1,2], Alessio Andronico PhD[1], Paolo Bosetti PhD[1], Juliette Paireau PhD[1,3], Lise Alter[4], Pierre-Yves Boëlle[5], Arnaud Fontanet[6,7], Daniel Lévy-Bruhl[3], Simon Cauchemez PhD[1]

1. Mathematical Modelling of Infectious Diseases Unit, Institut Pasteur, UMR2000, CNRS, Paris, France
2. Collège Doctoral, Sorbonne Université, Paris, France
3. Santé Publique France, French National Public Health Agency, Saint-Maurice, France
4. Haute Autorité de Santé, Saint-Denis la Plaine, France
5. Institut Pierre Louis d'Epidémiologie et de Santé Publique, Sorbonne Université, INSERM, Paris, France
6. Emerging Diseases Epidemiology Unit, Institut Pasteur, Paris, France
7. PACRI Unit, Conservatoire National des Arts et Métiers, Paris, France

This supplementary material is hosted by Eurosurveillance as supporting information alongside the rapid communication **Benefits and risks associated with different uses of the Vaxzevria vaccine: a modelling study**, on behalf of the authors, who remain responsible for the accuracy and appropriateness of the content. The same standards for ethics, copyright, attributions and permissions as for the article apply. Supplements are not edited by Eurosurveillance and the journal is not responsible for the maintenance of any links or email addresses provided therein.

Content

Supplementary material

Supplementary figures S1-S4

Supplementary tables S1-S4

Supplementary materials

We summarise here the modelling framework that is presented in Tran Kiem et al.[1] and on which we rely for this analysis.

Data sources

Hospitalization data

We use daily hospitalization data and death data reported in the SI-VIC database, the national exhaustive surveillance system describing the trajectories of COVID-19 patients admitted in French public and private hospitals. Available information includes age, hospitalization date, as well as outcome date and type. We restrict our analysis to general ward and intensive care units (ICU) patients and exclude patients hospitalized in psychiatric care, emergency care or long-term and rehabilitation care units.

B.1.1.7 data

We use data from Flash surveys, a series of six nationwide surveys (Flash #1 through Flash #6) performed between January 7th and March 30th, 2021 to describe the spread of variants in France.[2] From the third round, private and public diagnostic laboratories voluntarily participate in these studies by sending samples to sequencing platforms. From these surveys, we use the number of positive SARS-CoV-2 RT-PCR tests performed and the number of B.1.1.7 sequences among the positive samples. For model calibration, we removed the data from Flash#3 due to the small sample size.

Vaccination data

We use vaccination data from the VAC-SI database, the national exhaustive information system including the number of doses by age, vaccine brand and date of injection to account for past vaccine roll-out.[3] These data indicate that by May 7th, 25.7% of the Metropolitan French adult population has received a first vaccine dose.

Model parametrization

We developed a deterministic SEEIR model stratified by age similar to the one used in Salje et al.[4] and Andronico et al.[5] We extended the model to account for the emergence of B.1.1.7 and the progressive roll-out of vaccination in France. The metropolitan French population is divided into the following 13 age groups: [0-10), [10-18), [18-30), [30-40), [40-45), [45-50), [50-55), [55-60), [60-65), [65-70), [70-75), [75-80), ≥ 80. We assume that individuals aged 0-9 y.o. and 10-17 y.o are respectively 50% and 75% less susceptible compared to adults.[6,7] The model is implemented with the R software using the *odin* package.[8]

Transmission model with two strains

We consider a transmission model with two strains: the historical lineage that was circulating throughout 2020 in France and the variant B.1.1.7. Upon infection, susceptible individuals (S) move to the compartment $E1_{hist}$ or $E1_{B.1.1.7}$ depending o[9]n the strains they have been infected by. After an average duration of 4.0 days (same natural history of the disease for all lineages), infected individuals move to the E2 compartment ($E2_{hist}$ or $E2_{B.1.1.7}$) where they become infectious. They stay in this compartment for an average duration of 1.0 day before moving to

the I compartment (IM or IH for mild infections or infections requiring an admission in hospital) in which a fraction of them will develop symptoms. The average length of stay in I is equal to 3 days. The proportion of individuals that will require an admission in hospital is age and strain-dependent. We assume that individuals infected with B.1.1.7 strain have a 64% higher probability of being hospitalized.[10] For the historical lineages, we use age-specific probabilities of hospitalization estimated from the joint analysis of age-stratified serological and hospitalization data in France.[11] Finally, individuals in the Imild compartment will recover (R compartment), while individuals in the IH will move to the $\bar{I}H$ compartment before being admitted in hospital (compartment H). A schematic illustration of the model is depicted in Figure S4.

Transmissibility advantage of the B.1.1.7 strain

In our baseline scenario, we assume that the B.1.1.7 variant is 60% more transmissible than the historical virus (corresponding to the lineages circulating in France during 2020), consistent with what was estimated in France.[12] We consider an increased transmissibility of 40% as a sensitivity analysis. We define the intervention reproduction number RI as the average number of persons infected by a case under the set of control measures put in place if there was no immunity in the population. This number is available for the B.1.1.7 variant ($R_I^{B.1.1.7}$) and for the historical lineages (R_I^{hist}). The parameter α is the increased transmissibility associated with B.1.1.7. We then have the following relationship between the two reproduction numbers:

$$R_I^{B.1.1.7} = (1 + \alpha) * R_I^{hist}$$

We also assume that individuals infected with this strain have a 64% higher probability of being admitted to hospital than those infected with the historical virus.[13]

Epidemiological trajectories

The French government has announced that restrictive measures implemented since March 2021 would be progressively lifted from May 19th, 2021. In our baseline scenario, we assume that between May 19, 2021 and July 1st, 2021, the intervention reproduction number R_I of the historical virus is equal to 1.1. As sensitivity analyses, we explore values of 1.0 and 1.2. Between July 1st and September 1st, we assume that this value will increase to 1.3, as measured during the summer of 2020 when control measures were largely relaxed.[14] We use the intervention reproduction number R_I to capture the impact of climate or interventions on the transmission rate.

Accounting for the emergence of B.1.1.7

To calibrate our model, we utilize a two-step approach. We first fit a one-strain model to hospitalization data from March 2020 to January 2021, in order to obtain the state of the ordinary differential equations system on January 1st, 2021. We then use the latter as the initial condition to seed a second, two-strain, model that is calibrated from January 1st to May 14th, 2021. The proportion of individuals infected with the B.1.1.7 variant on January 1st is estimated along with the other free parameters (see below).

Accounting for vaccination

Vaccine prioritization

Before May 7th, 2021, we use vaccination data from the VAC-SI database, an information system describing doses distributed by date of injection. Information about the age of the individual receiving the dose is available and the status of the distributed dose (first or second) is available. From May 7th, 2021, we consider a prioritization strategy based on age with the following prioritization order: (i) over 75 y.o., (ii) 65-74 y.o., (iii) 55-64 y.o., (iv) 50-64 y.o. and (v) 18-49 y.o. The vaccination starts within a group when the target vaccine coverages are reached in groups of higher priority. We consider target vaccine coverages of 85% in individuals older than 65 y.o. and 70% in individuals aged 18-64 y.o.

Vaccination campaign characteristics

We consider a one-dose scheme for the Janssen vaccine and a two-doses distribution scheme for mRNA and Vaxzevria vaccines with a delay of 42 days between doses for mRNA vaccines and 84 days for Vaxzevria, in line with recommendations in France. We take into consideration the constraints associated with the vaccine delivery schedule (Table S2), the vaccination roll-out pace and the delay between doses. First doses are distributed when available, always ensuring that second doses (if required) will be available within the assumed delay. Following the prioritization defined above, this allows us to define the number of individuals vaccinated in the different age groups by date of injection. We assume that vaccine protection is acquired 15 days after administration of the first dose.

Assessing the impact of Vaxzevria distribution campaigns

Additional number of ICU admissions and deaths averted in the different age groups

Under each scenario regarding the distribution of Vaxzevria, we compute the number of hospitalizations expected between May 1st and September 1st, 2021 in the different age groups. We then compute the number of hospitalizations averted compared to a reference scenario regarding the distribution of the Vaxzevria vaccine (either used only in those older than 55 y.o. or not distributed in the adult population). From this, we derive estimates of the number of ICU admissions and deaths averted in the different age groups. We compute 95% confidence intervals for the probabilities of ICU admission and death after hospitalization in the different age groups (Figure S3, Table S3) using the outcomes of patients from the SI-VIC database admitted in hospital during March 2021 (case resampling with 1,000 bootstrap samples).

Expected number of TTS in the different age groups

We compute the expected number of TTS occurring in the different age groups These estimates were performed at a time where few individuals had received a second dose of Vaxzevria which made it difficult to assess the risk of TTS after the second dose. In our baseline scenario, we assume that risk estimated from the EMA would correspond to a risk per dose (i.e. that the risk of TTS is the same after the two doses). As a sensitivity analysis, we consider a scenario where no TTS occurs after the second dose.

Statistical framework

Model parameters are estimated using Markov Chain Monte Carlo (MCMC) sampling using flat, non-informative, priors. The likelihood function is given by:

$$L = \prod_i g_1(H_i^{obs} \mid H_i^{exp}) \cdot \prod_j g_2(N_j^{B.1.1.7}, N_j^{pos}, p_j^{B.1.1.7})$$

where g_1 is the density of a Poisson distribution, g_2 the density of a Binomial distribution, H_i^{obs} and H_i^{exp} the observed and expected total (all age groups) number of hospital admissions for day *i*, $N_j^{B.1.17}$ the observed number of B.1.1.7 sequences among the N_j^{pos} positive PCR tests on date *j*, and $p_j^{B.1.1.7}$ is the expected proportion B.1.1.7 infections - defined as the proportion of individuals in the $E2_{B.1.1.7}$ and $I_{B.1.1.7}$ compartments among all individuals in the E2 and I compartments - on date *j*.

The first term represents the contribution from hospitalization data so that the index *i* runs over all days from January 1st to May 14th, 2021. The second term represents instead the contribution from the observed proportion of B.1.1.7 among positive tests so that the index *j* runs over the dates of Flash#1 through Flash#6 surveys, excluding Flash#3. Model parameters and estimates are summarized in Table S4. The model fit to the data is represented in Figure S5.

Supplementary figures

Figure S1

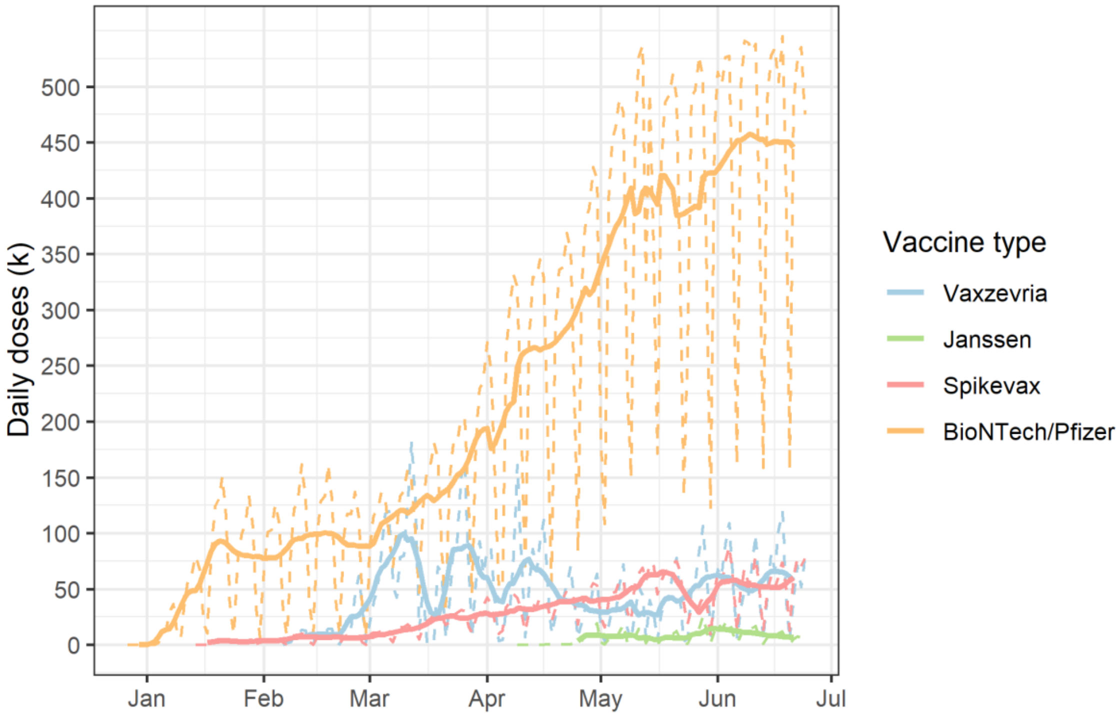

Figure S1: Number of doses (both first and second) administered by date of injection and stratified by vaccine type in France. The dashed lines correspond to daily data and the continuous lines correspond to the 7-day rolling average. These numbers come from data reported in the VAC-SI dataset (Data on individuals vaccinated against COVID-19).[15]

Figure S2

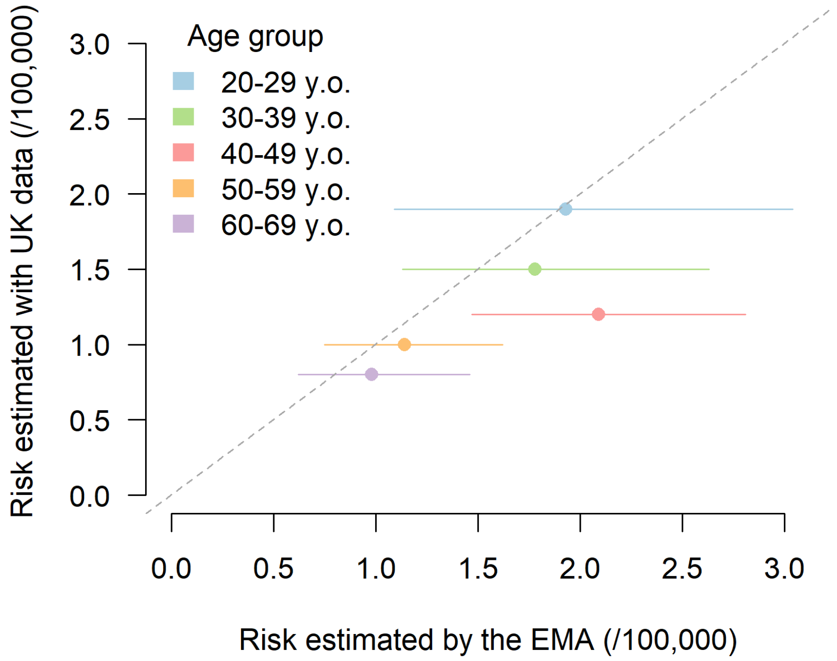

Figure S2: Comparison between risks of TTS estimated by the EMA and risks of specific blood clots estimated by the Winton Centre for Risk and Evidence Communication.[16] Risks computed by the Winton Centre for Risk and Evidence Communication are based on UK data up until April 28th, 2021. Horizontal lines correspond 95% confidence intervals for risks computed by the EMA.

Figure S3

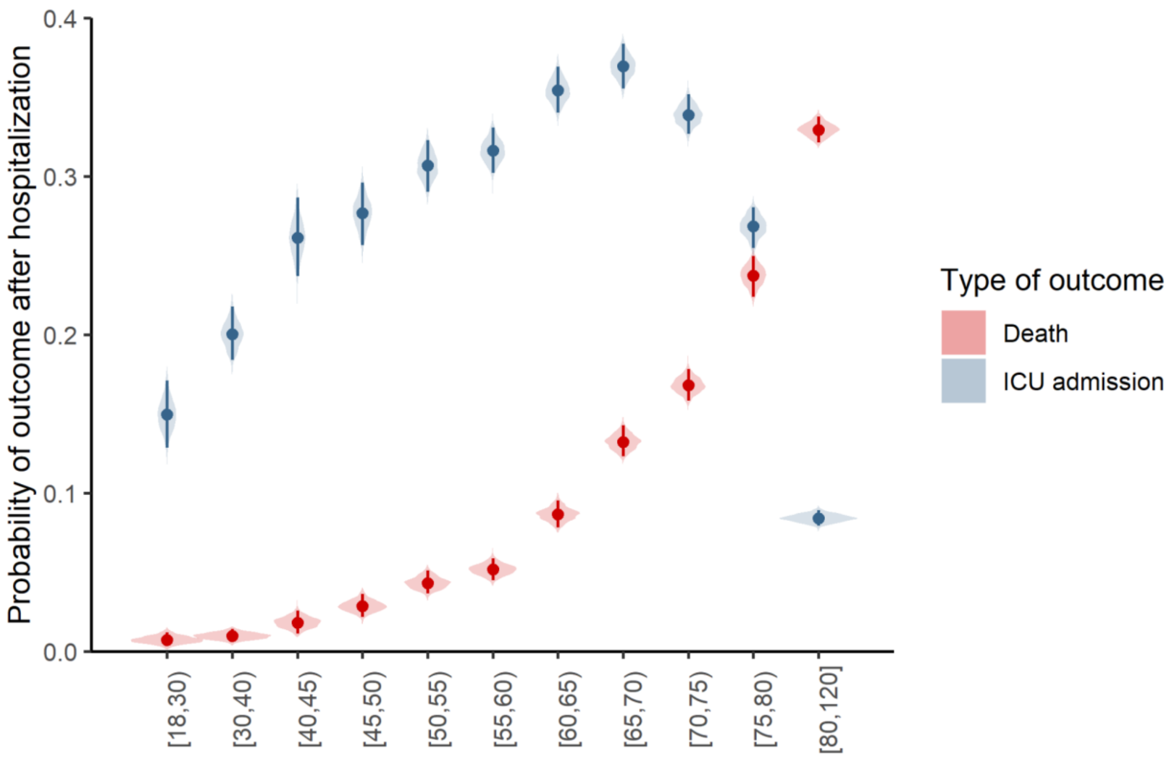

Figure S3: Probabilities of ICU admission and death given hospitalization in the different age groups. The ranges correspond to 95% confidence intervals derived from 1,000 bootstrap samples. Violin plots correspond to the distribution of the different samples drawn to derive these intervals.

Figure S4

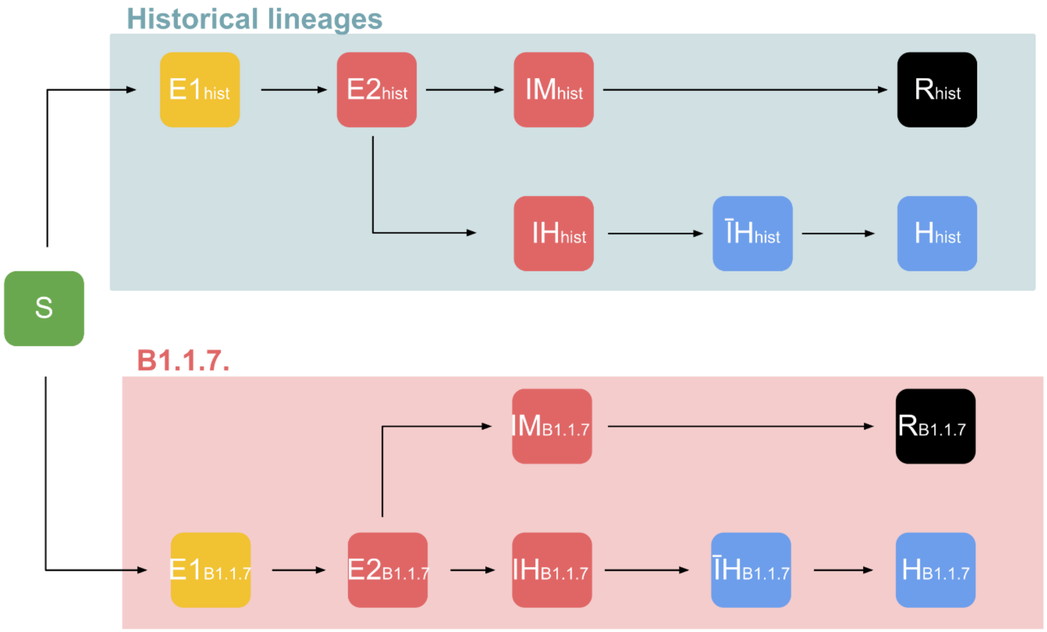

Figure S4: Schematic illustration of the transmission model accounting for the emergence of B.1.1.7 in France. See supplementary materials.

Figure S5

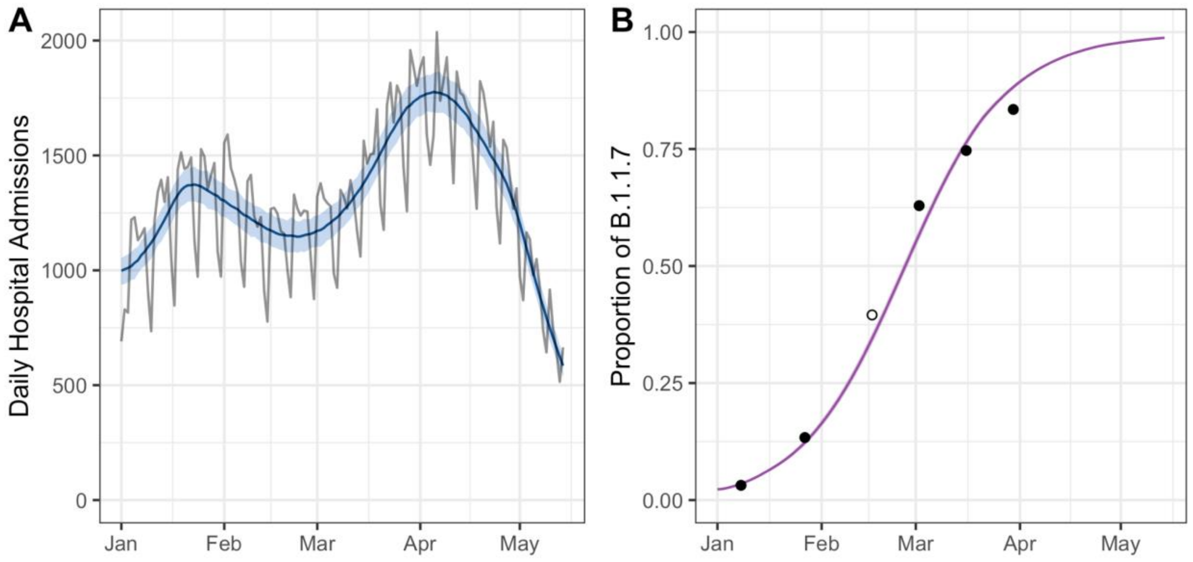

Figure S5. Fit to the data. (A) Daily hospital admissions through time. The blue line and area correspond to model posterior mean and 95% credible intervals, while the grey line corresponds to data. **(B) Proportion of B.1.1.7 through time.** The purple line and area correspond to model posterior mean and 95% credible intervals, black dots represent data used for calibration, while the white dot represents data not used for calibration (Flash#3).

Supplementary tables

Table S1: Incidence rates of TTS in vaccinated individuals 30 days post-vaccination computed by the EMA. Results are reported per 100,000.

Age group	Incidence rate
20-29 y.o.	1.93 (1.09 - 3.04)
30-39 y.o.	1.78 (1.13 - 2.63)
40-49 y.o.	2.09 (1.47 - 2.81)
50-59 y.o.	1.14 (0.75 - 1.62)
60-69 y.o.	0.98 (0.62 - 1.46)
70-79 y.o.	0.50 (0.16 - 1.12)
Over 80 y.o.	0.45 (0.09 - 1.25)
Total	1.34 (1.12 - 1.57)

Table S2: Delivery calendar used for doses allocation (in million doses). These numbers are computed based on a delivery calendar communicated by the French Ministry of Health on May 3rd, 2021.

	Pfizer/BioNTech	Moderna	Vaxzevria	Janssen
December 2021	0.5	0	0	0
January 2021	2.0	0.1	0	0
February 2021	2.5	0.4	1.6	0
March 2021	4.9	0.9	2.8	0
April 2021	8.1	1.0	3.0	0.4
May 2021	10.1	1.8	2.9	1.4
June 2021	19.8	2.4	4.4	5.7
July 2021	10.0	3.8	4.8	5.9
August 2021	10.0	5.7	4.8	5.9

Table S3: Probabilities of ICU admission and death given hospitalisation. These numbers are computed from the trajectory of COVID-19 patients admitted in metropolitan French hospitals during March 2021 (see Methods).

Age group	Probability of ICU admission after hospitalisation (95% confidence interval)	Probability of death after hospitalisation (95% confidence interval)
18-29 y.o.	0.15 (0.13 - 0.17)	0.007 (0.003 - 0.012)
30-39 y.o.	0.2 (0.18 - 0.22)	0.01 (0.006 - 0.014)
40-44 y.o.	0.26 (0.24 - 0.29)	0.01 (0.006 - 0.014)
45-49 y.o.	0.28 (0.26 - 0.3)	0.03 (0.02 - 0.04)
50-54 y.o.	0.31 (0.29 - 0.32)	0.04 (0.04 - 0.05)
55-59 y.o.	0.32 (0.3 - 0.33)	0.05 (0.04 - 0.06)
60-64 y.o.	0.35 (0.34 - 0.37)	0.09 (0.08 - 0.1)
65-69 y.o.	0.37 (0.36 - 0.38)	0.13 (0.12 - 0.14)
70-74 y.o.	0.34 (0.33 - 0.35)	0.17 (0.16 - 0.18)
75-79 y.o.	0.27 (0.25 - 0.28)	0.24 (0.22 - 0.25)
over 80 y.o.	0.08 (0.08 - 0.09)	0.33 (0.32 - 0.34)

Table S4: Model parameters and estimates. The estimates correspond to the baseline scenario (60% transmission advantage of B.1.1.7 with respect to the historical lineages, and vaccines 90% effective against severe disease and 80% effective against infection).

**Parameter**	**Estimate (Mean and 95% CI)**
Proportion of B.1.1.7 on January 1st, 2021	2.3% [2.2%, 2.4%]
B.1.1.7 intervention reproduction number R_I from January 1st to January 14th, 2021	2.19 [2.17, 2.21]
B.1.1.7 intervention reproduction number R_I from January 15th to January 31st, 2021	1.66 [1.64, 1.69]
B.1.1.7 intervention reproduction number R_I from February 1st to February 14th, 2021	1.61 [1.58, 1.64]
B.1.1.7 intervention reproduction number R_I from February 15th to February 28th, 2021	1.60 [1.56, 1.63]
B.1.1.7 intervention reproduction number R_I from March 1st to March 19th, 2021	1.60 [1.58, 1.62]
B.1.1.7 intervention reproduction number R_I from March 20th to April 4th, 2021	1.37 [1.35, 1.39]
B.1.1.7 intervention reproduction number R_I from April 5th to April 23rd, 2021	1.33 [1.31, 1.35]
B.1.1.7 intervention reproduction number R_I after April 24th, 2021	0.96 [0.93, 1.00]

References

1 Tran Kiem C, Massonnaud C, Levy-Bruhl D, *et al.* Short and medium-term challenges for COVID-19 vaccination: from prioritisation to the relaxation of measures. 2021; published online April 6. https://hal-pasteur.archives-ouvertes.fr/pasteur-03190243 (accessed May 27, 2021).

2 Santé Publique France. Enquêtes Flash : évaluation de la circulation des variants du SARS-CoV-2 en France. https://www.santepubliquefrance.fr/etudes-et-enquetes/enquetes-flash-evaluation-de-la-circulation-des-variants-du-sars-cov-2-en-france (accessed May 19, 2021).

3 Santé publique France. Données relatives aux personnes vaccinées contre la Covid-19. 2021; published online Jan 26. https://www.data.gouv.fr/fr/datasets/donnees-relatives-aux-personnes-vaccinees-contre-la-covid-19-1/ (accessed May 12, 2021).

4 Salje H, Tran Kiem C, Lefrancq N, *et al.* Estimating the burden of SARS-CoV-2 in France. *Science* 2020; 369: 208–11.

5 Andronico A, Tran Kiem C, Paireau J, *et al.* Evaluating the impact of curfews and other measures on SARS-CoV-2 transmission in French Guiana. *Nat Commun* 2021; 12: 1634.

6 Viner RM, Mytton OT, Bonell C, *et al.* Susceptibility to SARS-CoV-2 Infection Among Children and Adolescents Compared With Adults: A Systematic Review and Meta-analysis. *JAMA Pediatr* 2021; 175: 143–56.

7 Davies NG, CMMID COVID-19 working group, Klepac P, *et al.* Age-dependent effects in the transmission and control of COVID-19 epidemics. Nature Medicine. 2020; 26: 1205–11.

8 FitzJohn R. odin: ODE Generation and Integration. 2020 https://CRAN.R-project.org/package=odin (accessed May 19, 2021).

9 FitzJohn R. odin: ODE Generation and Integration. 2020 https://CRAN.R-project.org/package=odin (accessed May 19, 2021).

10 Bager P, Wohlfahrt J, Fonager J, *et al.* Increased Risk of Hospitalisation Associated with Infection with SARS-CoV-2 Lineage B.1.1.7 in Denmark. 2021; published online March 2. DOI:10.2139/ssrn.3792894.

11 Lapidus N, Paireau J, Levy-Bruhl D, *et al.* Do not neglect SARS-CoV-2 hospitalization and fatality risks in the middle-aged adult population. Infectious Diseases Now. 2021; 51: 380–2.

12 Gaymard A, Bosetti P, Feri A, *et al.* Early assessment of diffusion and possible expansion of SARS-CoV-2 Lineage 20I/501Y.V1 (B.1.1.7, variant of concern 202012/01) in France, January to March 2021. Eurosurveillance. 2021; 26. DOI:10.2807/1560-7917.es.2021.26.9.2100133.

13 Bager P, Wohlfahrt J, Fonager J, *et al.* Increased Risk of Hospitalisation Associated with Infection with SARS-CoV-2 Lineage B.1.1.7 in Denmark. 2021; published online March 2. DOI:10.2139/ssrn.3792894.

14 Santé Publique France. COVID-19 : point épidémiologique du 13 août 2020. {Santé Publique France}, 2020 https://www.santepubliquefrance.fr/maladies-et-traumatismes/maladies-et-infections-respiratoires/infection-a-coronavirus/documents/bulletin-national/covid-19-point-epidemiologique-du-13-aout-2020 (accessed May 28, 2021).

15 Santé publique France. Données relatives aux personnes vaccinées contre la Covid-19. [Data relating to people vaccinated against COVID-19].]. Saint-Maurice: Santé publique France. [Accessed: 29 Jun 2021]. French. Available from: https://www.data.gouv.fr/fr/datasets/donnees-relatives-aux-personnes-vaccinees-contre-la-covid-19-1

16 Winton Centre Cambridge. https://wintoncentre.maths.cam.ac.uk/news/latest-data-mhra-blood-clots-associated-astra-zeneca-covid-19-vaccine (accessed May 27, 2021).
